# Intraosseous blood aspirates analysed by a portable cartridge-based device

**DOI:** 10.1186/cc9558

**Published:** 2011-03-11

**Authors:** G Strandberg, A Larsson, M Lipcsey, M Eriksson

**Affiliations:** 1 Anesthesia & Intensive Care, Uppsala University, Sweden; 2 Clinical Chemistry, Uppsala University, Sweden

## Introduction

Intraosseous (IO) needles play an important role in medical emergencies, when venous access is difficult to establish. IO needles are suitable for infusion, but their use for blood sampling has been questioned, since aggregates of marrow substances may block analysers [1]. However, portable laboratory instruments have been developed, where the blood may be analysed within a separate cartridge. We decided to evaluate whether such a portable device is suitable for analysis of blood gases and electrolytes in aspirates obtained from IO needles during a 6-hour period. A second aim of this study was to compare such IO aspirates with arterial blood samples, both of them analysed by a handheld laboratory analysis system.

## Methods

IO needles (Im-Medico) were inserted bilaterally in the proximal tibiae of five anaesthetised healthy pigs. Blood gases and electrolytes (Na, K, Ca) were taken hourly. IO aspirates and arterial blood samples were immediately analysed by an i-STAT handheld (Abbott Point of Care) equipped with EG7+ and CG4+ cartridges. A coefficient of variance (CV) >20%, was regarded as the upper limit of quantification [2]. Bland-Altman curves were used to assess agreement between the two methods [3].

## Results

Repeated IO aspirates were easily obtained during the entire 6-hour period. There were excellent consistencies in blood gases and electrolytes, between IO aspirates from the left and right tibiae, except for BE, where CV >20%. IO aspirates were compared with arterial samples. There were compliant values between these sources regarding electrolytes, Hb, pH, pCO_2 _and SBC. This was in contrast to BE, lactate, PO_2 _and SO_2_, which all exhibited CV >20%. Although both SO_2 _and PO_2 _were higher in arterial samples as compared with IO samples, there were high correlations between these two variables in arterial blood and IO aspirates (*R *> 0.9; *P *< 0.001 and *R *= 0.7; *P *< 0.001, respectively). There were only minor changes over time in any of these variables during the entire experimental period.

## Conclusions

Blood gases and electrolytes in IO blood aspirates are easily analysed by a handheld device during a 6-hour period. The development of this cartridge-based laboratory analysis system strengthens the concept of using IO needles as a valuable tool in medical emergency situations. If blood gases are to be evaluated in IO aspirates, SBC seems to reflect arterial conditions better than BE does.
